# Nature-Based Mental Health - what kind of interventions is the best?

**DOI:** 10.1192/j.eurpsy.2023.1911

**Published:** 2023-07-19

**Authors:** M. Gawrych

**Affiliations:** Institute of Psychology, The Maria Grzegorzewska University, Warsaw, Poland

## Abstract

**Introduction:**

Mental health deteriorated worldwide during the COVID-19 pandemic. The healthcare sector recognises the role of nature in mental health. Passive and active interactions with nature reduce stress, anxiety, and depression. Theoretical frameworks for mental health benefits from nature interventions include medical, evolutionary, relational, eco-psychological and human activity perspctives.

**Objectives:**

To assess the usefulness of the nature-based interventions in relation to well-being improvement.

**Methods:**

The six-month survey was carried out in a forest in the administrative district of the capital city of Warsaw.Over 70 adult volunteers took part in structured sessions of active nature-based interventions, lasting ca. 1.5 hour.The forest bathing methodology was based on the review literature. Participants anonymously filled in on-line semi-structured questionnaire containing GHQ-30, DASS-21 andlife satisfaction. questionnaire. Between sessions, participants were asked toassessment their mood profile and life satisfaction. After every session they assessed particular interventions in terms of usefulness on 5-point Likert scale.

**Results:**

The authors will present the results of the study and key findings.

**Conclusions:**

It is expected that the study may provide a significant contribution to the knowledge’ development about the nature-based interventions. In particular, we can learn about the impact of several interventions (active exercise, visualization, mindful walking, mindfulness perception) on improving the well-being of participants.

**Disclosure of Interest:**

None Declared

